# Outcomes of Patients With Prior Biologic Intolerance Are Better Than Those With Biologic Failure in Clinical Trials of Inflammatory Bowel Disease

**DOI:** 10.1093/ecco-jcc/jjae151

**Published:** 2024-09-20

**Authors:** Sunil Samnani, Emily C L Wong, Hasan Hamam, Parambir S Dulai, John K Marshall, Vipul Jairath, Walter Reinisch, Neeraj Narula

**Affiliations:** Department of Medicine, Division of Gastroenterology, McMaster University, Hamilton, ON, Canada; Farncombe Family Digestive Health Research Institute, McMaster University, Hamilton, ON, Canada; Department of Medicine, Division of Gastroenterology, McMaster University, Hamilton, ON, Canada; Farncombe Family Digestive Health Research Institute, McMaster University, Hamilton, ON, Canada; Department of Medicine, Division of Gastroenterology, McMaster University, Hamilton, ON, Canada; Farncombe Family Digestive Health Research Institute, McMaster University, Hamilton, ON, Canada; Division of Gastroenterology, Northwestern University, Chicago, IL, USA; Department of Medicine, Division of Gastroenterology, McMaster University, Hamilton, ON, Canada; Farncombe Family Digestive Health Research Institute, McMaster University, Hamilton, ON, Canada; Division of Gastroenterology, Department of Medicine, Western University, London, ON, Canada; Department of Internal Medicine III, Division of Gastroenterology and Hepatology, Medical University of Vienna, Währinger Gürtel 18-20, Vienna, Austria; Department of Medicine, Division of Gastroenterology, McMaster University, Hamilton, ON, Canada; Farncombe Family Digestive Health Research Institute, McMaster University, Hamilton, ON, Canada

**Keywords:** Inflammatory bowel disease, biologic intolerance, biologic failure, clinical outcomes

## Abstract

**Background and Aims:**

Inflammatory bowel disease (IBD) trials often stratify patients by prior biologic exposure, including prior biologic failure or intolerance. This study aimed to assess clinical outcomes in IBD patients with prior biologic failure vs intolerance treated with ustekinumab or vedolizumab.

**Methods:**

A post-hoc analysis of ulcerative colitis (UC) and Crohn’s disease (CD) clinical trials for ustekinumab (UNITI and UNIFI) and vedolizumab (GEMINI-1 and GEMINI-2) was performed. Clinical response, clinical remission, and endoscopic improvement (for UC) were compared among biologic naïve, biologic failure, and biologic intolerant patients. Statistical analyses, including chi-square tests and logistic regression, were performed.

**Results:**

A total of 1178 UC and 1439 CD patients received either ustekinumab or vedolizumab. In UC, biologic intolerant patients exhibited higher clinical response (54.7% vs 38.8%, aOR 1.87 [95% CI, 0.93-3.73]), clinical remission (25.0% vs 11.0%, aOR 2.84 [95% CI, 1.47-5.49]), and endoscopic improvement (40.6% vs 24.8%, aOR 2.76 [95% CI, 1.28-5.94]) compared to biologic failure, with outcomes similar to biologic naïve patients. In biologic intolerant CD patients, clinical response was similar between prior biologic failure and intolerance (34.2% vs 32.8%), but after adjustment for potential confounders, biologic intolerance was associated with higher odds of clinical response (aOR: 1.67, 95% CI, 1.09-2.55), with no significant difference observed for clinical remission (aOR: 1.48, 95% CI, 0.88-2.49).

**Conclusions:**

Improved treatment outcomes were generally observed in patients with biologic intolerance compared to failure, especially in UC, where outcomes were similar to biologic naïve patients. Future clinical trials should meticulously differentiate prior biologic failure vs intolerance to mitigate potential bias.

## Graphical Abstract

**Graphical Abstract:**
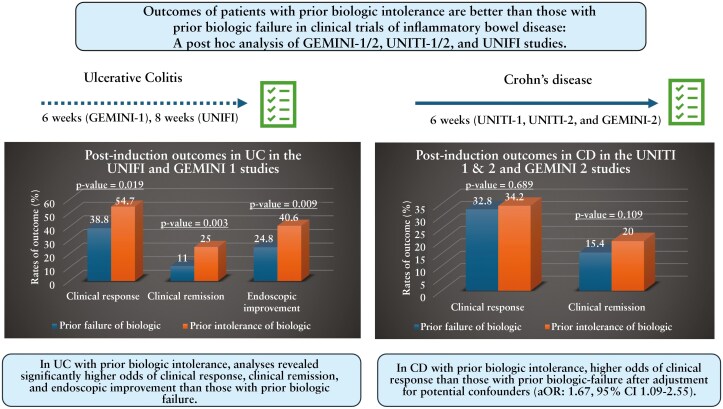

## 1. Introduction

Inflammatory bowel diseases (IBDs), encompassing ulcerative colitis (UC) and Crohn’s disease (CD), are chronic inflammatory conditions affecting the gastrointestinal tract. The advent of biologic therapies has revolutionized IBD treatment by reducing the need for steroid therapy, promoting mucosal healing, and decreasing hospitalizations and surgeries, thereby significantly enhancing the quality of life for patients.^1^ Despite their efficacy, a substantial proportion of patients do not respond or lose response to their initial biologic therapy and are considered biologic failures. Further, some patients may respond to therapy but experience adverse events and are considered to have an intolerance to a prior biologic.^2^

Randomized placebo-controlled trials have commonly observed that patients with prior biologic use typically represent a cohort that is harder to treat, and often have reported lower efficacy rates for clinical and endoscopic outcomes compared to biologic naïve patients.^3–6^ However, it is unclear whether all patients who have previously been exposed to biologics should be grouped into one cohort. For example, a patient doing well on biologic A but developed a rash and then had to switch to biologic B, is a different scenario to a patient who has primary nonresponse to biologic A and had to switch to biologic B; nonetheless, in the clinical trial scenario, both patients would be considered in the same category of prior biologic intolerance or failure. To our knowledge, no studies have explored whether clinical outcomes are different in patients with prior biologic failure compared to those with intolerance.

The primary aim of this study was to explore whether clinical outcomes in IBD patients in clinical trials of ustekinumab or vedolizumab differ according to whether they had prior biologic failure vs prior biologic intolerance.

## 2. Methods

### 2.1. Study Design

Patient-level data from randomized, placebo-controlled clinical trials UNITI-1 (NCT01369329), UNITI-2 (NCT01369342),^3^ UNIFI (NCT02407236),^4^ GEMINI-1 (NCT00783718),^5^ and GEMINI-2 (NCT00783692)^6^ were utilized. Data access and permission for analyses were acquired through VIVLI (Cambridge, MA; Protocol #00009332) by permission from Takeda, Inc. (Deerfield, IL) and through the YODA (Yale Open Data Access # 2023-5300) Project and by permission from Janssen Inc. The Hamilton Integrated Research Ethics Board determined that a local ethics review was not needed as data were previously collected and de-identified.

### 2.2. Participants

Design and eligibility criteria for the UNIFI, GEMINI-1 and 2, and UNITI-1 and 2 studies have previously been published.^3–6^ For this study, patients were classified based on their prior experience with biologics. Patients with prior failure were considered those who did not have adequate symptomatic or endoscopic improvement with one previous biologic, or lost response after an initial clinical response (usually anti-TNF). Patients with intolerance to biologics had experienced treatment-related toxicity such as an infusion-related reaction, psoriasiform skin lesion, demyelination, congestive heart failure, infection, or other clinically meaningful adverse events that required discontinuation of the biologic therapy. The biologic naïve population comprised patients who had never received biological treatment. For the purpose of this analysis, the biologic failure and intolerance cohorts were restricted to patients who had one prior failure or intolerance, and patients with multiple failures or intolerances were excluded.

### 2.3. Clinical Outcomes

Our primary objective was to evaluate the clinical response and remission at postinduction (for CD trials, this occurred at week 6 for all of UNITI-1, UNITI-2, and GEMINI-2, and for UC, it was evaluated at Week 6 in GEMINI-1 and Week 8 in UNIFI). For UC, clinical response was characterized by a reduction in the total Mayo Score by at least 3 points and by 30% or more compared to the baseline. This reduction must be accompanied by a decrease in the rectal bleeding subscore of at least 1 point or an absolute rectal bleeding subscore of 0 or 1. Clinical remission for UC was defined as achieving a Mayo score of 2 points or less, with no individual subscore exceeding 1 point. Endoscopic improvement was attaining an endoscopic Mayo subscore of 0 or 1.

For CD, clinical response was defined as a decrease in the Crohn’s Disease Activity Index (CDAI) of at least 100 points compared to the baseline. Clinical remission was defined as achieving a CDAI score of 150 or lower. Endoscopic evaluation was not routinely conducted at postinduction within the CD trials, so it was not assessed in this study. The independent variable was based on patients’ prior biologic status: prior intolerance to one biologic, prior failure of one biologic, or biologic naïve.

### 2.4. Statistical Analyses

The primary analysis was conducted as intention-to-treat, where patients with missing data were assumed to not have achieved the outcomes of interest. Continuous variables are presented as means (SD) or as medians (interquartile ranges [IQR]), and dichotomous variables are presented as proportions or percentages. Descriptive statistics are used to summarize outcomes of interest such as clinical response and remission between groups. Cochran-Mantel-Haenszel chi-square test, with a significance level of 0.05, was used to compare dichotomous endpoints (i.e. clinical response, clinical remission, and endoscopic improvement) among treatment groups. Logistic regression models were used to compare postinduction outcomes among patients who were previously biologic intolerant and biologic naïve relative to those with biologic failure. Univariate analyses were conducted to evaluate the relationship between baseline variables and achievement of postinduction clinical remission. Subsequently, multivariate logistic regression using backward stepwise selection was performed and only those baseline variables with *p* < 0.05 on univariate analysis were considered for inclusion. Unadjusted odds ratios (ORs) and adjusted ORs (aORs) are presented with associated 95% CIs. Data were analyzed using Stata IC version 15.0 (Stata-Corp LLC, College Station, TX) available on the VIVLI secure platform.

## 3. Results

### 3.1. Baseline Characteristics of Patients With UC and CD Stratified by Prior Biologic Exposure

Baseline data from 1178 UC patients and 1439 CD patients were analyzed and summarized in Tables 1 and 2, respectively. Among UC patients, 66.8% (*n* = 787) were biologic naive, 27.8% (*n* = 327) had one prior biologic failure, and 5.4% (*n* = 64) had one prior biologic intolerance. Across different treatment history groups, the mean age of UC patients ranged from approximately 38.6-40.9 years. A higher proportion of males were observed in the prior failure group (66.7%) compared to the prior intolerance group (42.6%) and biologic naive group (58.9%). The majority of UC patients in all groups were of white race, with percentages ranging from 78.1% to 91%. Smoking history was reported in 18.8% of biologic naive patients, with lower percentages in the other groups. Disease duration varied, with the lowest median observed in biologic naive patients (3.9 years, IQR 7) compared to those with prior failure (4.8 years, IQR 5.8) and prior intolerance (6.1 years, IQR 7.8). Disease activity, as indicated by the Total Mayo Score, was slightly higher in patients with prior failure compared to those with prior intolerance and biologic naive status. The baseline characteristics of UC patients were further stratified by treatment with Vedolizumab or Ustekinumab. The baseline characteristics were similarly distributed between both ustekinumab and vedolizumab trials, with small differences observed in the distribution of gender, race, smoking, disease duration, and severe disease (Endoscopic Mayo score of 3; Supplementary Table 1).

**Table 1:** Baseline characteristics for UC patients (*n* = 1178).

Variable	Prior failure of 1 biologic	Prior intolerance of 1 biologic	Biologic naive
Total number of patients	327 (27.8)	64 (5.4)	787 (66.8)
Age in years, mean (SD)	40.8 (14.3)	38.6 (12.8)	40.9 (13.4)
Sex male, *n* (%)	218 (66.7)	27 (42.6)	464 (58.9)
Race White, *n* (%)	298 (91)	56 (87)	615 (78.1)
Smoking, *n* (%)	27 (8.3)	11 (17)	148 (18.8)
Disease duration years, median (IQR)	4.8 (5.8)	6.1 (7.8)	3.9 (7)
Vedolizumab, *n* (%)	233 (19.8)	58 (5)	421 (35.7)
Ustekinumab, *n* (%)	94 (8)	6 (0.5)	366 (31)
CRP mg/L, median (IQR)	4.5 (10.3)	7.8 (7.9)	5.1 (11.1)
Albumin g/L, mean (SD)	37.6 (5.2)	39.4 (4.3)	40.4 (5)
Total Mayo score, mean (SD)	8.7 (1.7)	7.9 (1.6)	8.51 (1.7)
Endoscopic Mayo score 3, *n* (%)	225 (68.8)	35 (55.3)	476 (60.5)

**Table 2:** Baseline characteristics for CD patients (*n* = 1439).

Variable	Prior failure of 1 biologic	Prior intolerance of 1 biologic	Biologic naive
Total number of patients	650 (45.2)	225 (15.6)	564 (39.2)
Age in years, mean (SD)	35.3 (12)	37.1 (11.3)	35.9 (13.5)
Sex male, *n* (%)	282 (46)	54 (24)	274 (48.7)
Race White, *n* (%)	614 (94.5)	209 (93.1)	493 (87.4)
Smoking, *n* (%)	296 (45.5)	73 (32.3)	168 (29.7)
Disease duration Years, median (IQR)	7.7 (7.4)	9.2 (9)	4.7 (7.6)
Vedolizumab, *n* (%)	458 (31.8)	161 (11.2)	348 (24.2)
Ustekinumab, *n* (%)	192 (13.4)	64 (4.4)	216 (15)
CRP mg/L, median (IQR)	8.5 (17.3)	9 (18.5)	8.89 (20)
Albumin g/L, mean (SD)	35.2 (5.4)	35.5 (4.9)	35.4 (5.5)
CDAI Score, mean (SD)	323.2 (62.9)	313.3 (58.7)	307.2 (58.9)

Similar trends were observed in CD patients, with 39.2% (*n* = 564) being biologic naive, 45.2% (*n* = 650) having one prior biologic failure, and 15.6% (*n* = 225) having one prior biologic intolerance. The mean age of CD patients ranged from approximately 35.3-37.1 years across different treatment groups. A higher percentage of males were observed in the biologic naive group (48.7%) compared to the prior failure group (46%) and the prior intolerance group (24%). Past or current smoking history was reported in 29.7% of biologic naive patients, with higher percentages in the other groups. Disease duration varied, with the lowest median observed in biologic naive patients (4.7 years, IQR 7.6) compared to those with prior failure (7.7 years, IQR 7.4) and prior intolerance (9.2 years, IQR 9). CRP and albumin levels were similar across treatment groups. The mean Crohn’s Disease Activity Index (CDAI) scores showed slight variations but were generally comparable, with the highest score observed in the prior failure cohort (323.2, SD 62.9), followed by the prior intolerance cohort (313.3, SD 58.7) and biologic naive cohort (307.2, SD 58.9). The baseline characteristics of CD patients were further stratified based on treatment within the vedolizumab or ustekinumab trials, and generally, the distribution of the baseline characteristics was comparable between the trials (Supplementary Table 2).

### 3.2. Outcomes in Patients With UC at Postinduction

Table 3 reveals clinical outcomes postinduction among UC patients treated with ustekinumab or vedolizumab in the UNIFI^4^ and GEMINI-1^5^ studies. The analysis revealed significant differences in clinical response, clinical remission, and endoscopic improvement between UC patients with prior failure and intolerance to biologic therapy (*p* < 0.05). Notably, patients with prior intolerance exhibited higher rates compared to those with biologic failure in terms of clinical response (54.7% vs 38.8%, *p* = 0.019), clinical remission (25% vs 11%, *p* = 0.003), and endoscopic improvement (40.6% vs 24.8%, *p* = 0.009). Biologic naive UC patients demonstrated similar outcomes to biologic intolerant patients, with clinical response, clinical remission, and endoscopic improvement rates of 52.9%, 19.8%, and 36.2%, respectively. When stratifying those with prior biologic failure into primary and secondary nonresponders, the intolerance group showed significantly better outcomes than both of these groups. Primary and secondary nonresponders to a prior biologic generally had similar rates of clinical response (40.8% vs 37.1%), clinical remission (10.8% vs 11.2%), and endoscopic improvement (22.9% vs 26.5%; Supplementary Table 3).

**Table 3:** Outcomes at postinduction among patients with ulcerative colitis treated with ustekinumab or vedolizumab in the UNIFI and GEMINI I studies.

Outcome	Prior failure of 1 biologic (*n* = 327)	Prior intolerance of 1 biologic (*n* = 64)	Biologic naïve (*n* = 787)	*p*-value (failure vs intolerance)	*p*-value (3 groups)
Postinduction clinical response^*^	127/327 (38.8)	35/64 (54.7)	416/787 (52.9)	0.019	<0.001
Postinduction clinical remission^**^	36/327 (11.0)	16/64 (25.0)	156/787 (19.8)	0.003	<0.001
Postinduction endoscopic improvement^***^	81/327 (24.8)	26/64 (40.6)	285/787 (36.2)	0.009	<0.001

*p* < 0.05 is significant.

^*^Reduction in Mayo score by at least 3 points and a decrease of at least 30% from baseline, with an accompanying decrease in the rectal bleeding subscore of at least 1 point or an absolute rectal bleeding subscore of 0 or 1.

^**^Mayo score of 2 or lower and no subscore higher than 1.

^***^MES ≤ 1.

In the univariate analysis of baseline variables, sex, race, biologic failure or intolerant history, albumin, total Mayo score, and endoscopic Mayo score had statistically significant associations with postinduction clinical remission (Supplementary Table 4). These variables were considered for inclusion in the multivariate model, where sex, biologic failure, albumin, and total Mayo score were selected based on backward stepwise selection. For adjusted ORs, sex, albumin, and total Mayo score were included.

Among biologic naive patients, both unadjusted and adjusted analyses revealed significantly higher odds of achieving clinical response (OR 1.58, 95% CI, 1.18-2.12; aOR 1.81, 95% CI, 1.24-2.64), clinical remission (OR 1.98, 95% CI, 1.24-3.15; aOR 3.57, 95% CI, 1.41-9.04), and endoscopic improvement (OR 2.73, 95% CI, 1.91-3.89; aOR 3.29, 95% CI, 2.06-5.25) compared to patients with prior biologic failure (Table 4). Similarly, among patients with prior biologic intolerance, unadjusted and adjusted analyses revealed higher odds of achieving clinical response (OR 1.76, 95% CI, 1.02-3.06; aOR 1.87, 95% CI, 0.93-3.73), clinical remission (OR 3.55, 95% CI, 1.79-7.08; aOR 2.84, 95% CI, 1.47-5.49), and endoscopic improvement (OR 3.1, 95% CI, 1.72-5.59; aOR 2.76, 95% CI, 1.28-5.94) compared to patients with prior biologic failure (Table 4). Similar trends were noted when examining the UNIFI and GEMINI-1 studies individually (Supplementary Tables 5 and 6).

**Table 4:** Logistic regression models for outcomes at postinduction among ustekinumab and vedolizumab UC patients who were biologic naïve or prior biologic intolerance compared to biologic failure.

	Biologic naïve				Biologic intolerance			
Outcome at postinduction (reference group: biologic failure)	Unadjusted OR (95% CI)	*p*-value unadjusted	Adjusted OR (95% CI)	*p*-value adjusted^*^	Unadjusted OR (95% CI)	*p*-value unadjusted	Adjusted OR (95% CI)	*p*-value adjusted^*^
Clinical response, *n* (%)^**^	1.58 (1.18-2.12)	0.002^*^	1.81 (1.24-2.64)	0.002^*^	1.76 (1.02-3.06)	0.043^*^	1.87 (0.93-3.73)	0.077
Clinical remission, *n* (%)^***^	1.98 (1.24-3.15)	0.004^*^	3.57 (1.41-9.04)	0.007^*^	3.55 (1.79-7.08)	<0.001^*^	2.84 (1.47-5.49)	0.002^*^
Endoscopic improvement, *n* (%)^****^	2.73 (1.91-3.89)	<0.001^*^	3.29 (2.06-5.25)	<0.001^*^	3.1 (1.72-5.59)	<0.001^*^	2.76 (1.28-5.94)	0.009

^*^Adjusted for baseline albumin, baseline total Mayo score and sex.

^**^Reduction in Mayo score by at least 3 points and a decrease of at least 30% from baseline, with an accompanying decrease in the rectal bleeding subscore of at least 1 point or an absolute rectal bleeding subscore of 0 or 1.

^***^Mayo score of 2 or lower and no subscore higher than 1.

^****^MES ≤ 1.

### 3.3. Outcomes in CD Patients at Postinduction

Table 5 reveals clinical outcomes postinduction among CD patients treated with ustekinumab or vedolizumab in the UNITI-1 & 2^3^ and GEMINI-2^6^ studies. There were no significant differences in postinduction clinical response or clinical remission between CD patients with prior failure and intolerance to biologic therapy at postinduction. Patients with prior intolerance exhibited numerically higher rates of clinical response (34.2%) and clinical remission (20%) compared to those with prior failure (32.8% and 15.4%%, respectively). Comparing the 3 groups revealed significant differences in all clinical outcomes in CD patients (*p* < 0.001), largely due to improved outcomes in the biologic naïve patients. As shown in Supplementary Table 7, there were no significant differences observed between primary and secondary nonresponders to previous biologic therapy in Week 6 clinical response (~35.1% and 29.6%, respectively) and clinical remission rates (15.9% and 14.6%).

**Table 5:** Outcomes at week 6 among patients with Crohn’s disease treated with ustekinumab or vedolizumab in the UNITI-1 and 2 and GEMINI-2 studies.

Outcome	Prior failure of 1 biologic (*n* = 650)	Prior intolerance of 1 biologic (*n* = 225)	Biologic naïve (*n* = 564)	*p*-value (failure vs intolerance)	*p*-value (3 groups)
Week 6 clinical response^*^	213/650 (32.8)	77/225 (34.2)	284/564 (50.4)	0.689	<0.001
Week 6 clinical remission^**^	100/650 (15.4)	45/225 (20.0)	174/564 (30.9)	0.109	<0.001

*p* < 0.05 is significant.

^*^Reduction in CDAI of at least 100 points from baseline.

^**^CDAI ≤ 150.

In the univariate analysis of baseline variables, race, disease duration, treatment received, biologic failure or intolerant history, C-reactive protein (CRP), albumin, and CDAI score had statistically significant associations with postinduction clinical remission (Supplementary Table 8). These variables were considered for inclusion in the multivariate model, where disease duration, treatment, CRP, albumin, and CDAI score were selected based on backward stepwise selection. For adjusted ORs, race, albumin, CRP, disease duration, treatment, and CDAI score were included.

Among biologic naive patients, both unadjusted and adjusted analyses revealed significantly higher odds of achieving clinical response (OR 3.59, 95% CI, 2.57-5.03; aOR 2.04, 95% CI, 1.43-2.93) and clinical remission (OR 2.17, 95% CI, 1.43-3.29; aOR 1.73, 95% CI, 1.11-1.68) compared to patients with prior biologic failure (Table 6). However, among patients with prior biologic intolerance, unadjusted and adjusted analyses revealed significantly higher odds of achieving clinical response (OR 1.74, 95% CI, 1.0-2.69; aOR 1.67, 95% CI, 1.09-2.55), but not for clinical remission (OR 1.38, 95% CI, 0.8-2.36; aOR 1.48, 95% CI, 0.88-2.49) compared to patients with prior biologic failure (Table 6). Similar trends were noted when comparing the 3 cohorts within the GEMINI-2 and UNITI-1 & 2 studies individually (Supplementary Tables 9 and 10). The median (IQR) disease duration was generally longer in CD patients with biologic intolerance as compared to those who were biologic naïve or biologic failures (Supplementary Table 11).

**Table 6:** Logistic regression models for outcomes at week 6 among ustekinumab and vedolizumab CD biologic naïve or prior biologic intolerance patients compared to those with biologic failure.

Outcome at Week 6 (reference group: biologic failure)	Biologic naïve				Biologic intolerance			
	Unadjusted OR (95% CI)	*p*-value unadjusted	Adjusted OR (95% CI)	*p*-value adjusted^*^	Unadjusted OR (95% CI)	*p*-value unadjusted	Adjusted OR (95% CI)	*p*-value adjusted^*^
Clinical response, *n* (%)^**^	3.59 (2.57-5.03)	<0.001^*^	2.04 (1.43-2.93)	<0.001^*^	1.74 (1.0-2.69)	0.185	1.67 (1.09-2.55)	0.017^*^
Clinical remission, *n* (%)^***^	2.17 (1.43-3.29)	<0.001^*^	1.73 (1.11-2.68)	0.015^*^	1.38 (0.8-2.36)	0.248	1.48 (0.88-2.49)	0.137

^*^Adjusted for baseline albumin, crp, disease duration, treatment, baseline CDAI score and race.

^**^Reduction in CDAI of at least 100 points from baseline.

^***^CDAI ≤ 150.

## 4. Discussion

For clinical trials in IBD, patients who have experienced prior biologic intolerance or failure are usually combined within one cohort.^7^ Our patient-related results provide a previously unaddressed, but relevant nuance to the crucial role of prior biologic exposure status plays a crucial role in determining treatment outcomes. While it has been established in the pivotal trials of vedolizumab and ustekinumab that biologic naive patients are demonstrating higher rates of clinical response and remission compared to those with prior biologic failure or intolerance in the pivotal trials of vedolizumab and ustekinumab. However, on assessing biologic intolerance in relation to treatment outcomes, we observed that patients who had previously experienced biologic intolerance have improved clinical and endoscopic postinduction outcomes in UC relative to those with prior biologic failure and improved clinical response in CD.

Previous studies showed higher clinical outcome rates for IBD patients with prior intolerance of biologics compared to those with treatment failure, aligning with our results.^8,9^ One hypothesis to explain this might be that patients with prior intolerance often responded positively to the treatment but had to stop it due to adverse events.^10^ Although intolerable side effects would preclude continued treatment, intolerance itself does not necessarily represent failed efficacy.^11^ This suggests that UC patients with prior biologic intolerance might not be as difficult to treat as patients with prior biological failure, who likely have more refractory disease.^12–14^ That was very evident for postinduction endoscopic improvement, where those with prior intolerance had almost 3-fold odds to achieve this compared to those with prior biologic failure. Therefore, grouping patients with biologic failure and intolerance in one cohort to compare them with the biologic naïve group for large trials may not be prudent to compare the outcomes. Stratification at clinical trial entry is often performed based on the history of biologic exposure, but combining biologic failures with intolerant patients could lead to an uneven distribution of patients with prior intolerance between trial arms, which has the potential to influence treatment results negatively or positively depending on where those patients are allocated.

The efficacy of a second biologic treatment in CD patients largely depends on the cause for switching, whether it was due to intolerance or failure.^15^ It has been shown that the clinical outcomes are improved when the reason to withdraw the first biologic was due to intolerance compared with failure.^15,16^ In our study, the outcomes of patients with CD treated with ustekinumab or vedolizumab in the UNITI-1 and 2 and GEMINI-2 studies showed no significant difference between biologic failure vs biologic intolerant groups. However, disease duration was generally longer among biologic intolerant patients compared to those with biologic failure. Longer disease duration has been associated with a lower likelihood of clinical and endoscopic improvement in CD, so it remains possible that the longer disease duration among biologic intolerant patients may have led to lower rates of improvement in this cohort.^12,17^ Indeed, in multivariate analyses that adjusted for disease duration among other variables, clinical response at Week 6 was significantly higher among biologic intolerant patients compared to biologic failures.

In contrast, significant differences in clinical outcomes were observed in the UC analyses, with improved outcomes among those with prior biologic intolerance compared to biologic failure, despite longer disease duration seen among those with prior biologic intolerance (median 6.1 vs 4.8 years). Disease duration is not thought to be as relevant to outcomes in UC as compared to CD.^18^ Although, unclear why disease duration differed significantly, it is possible that patients with biologic intolerance had responded to therapy and been on therapy for a number of months or years prior to developing an intolerance. Another potential explanation to explain differences observed in UC between those with prior biologic failure compared to intolerance could be fewer patients with severe disease (endoscopic Mayo score of 3) in the intolerance group; however, these differences in outcomes remained significant in the multivariate analyses including adjustment for total Mayo score.

To the best of our knowledge, this is the first study to use individual patient-level data and after adjusting for potential confounders has demonstrated a significant difference in outcomes of efficacy of advanced therapies in IBD patients comparing patients with prior intolerance of one biologic to those with a biologic failure. Multiple high-quality, prospectively collected phase 3 clinical trial datasets were used for this post-hoc analysis. However, it is important to acknowledge the limitations of our study. The sample size of the prior intolerance to one biologic group for UC was small when compared to the other cohorts. Secondly, we present the results of multiple comparisons, and there remains a chance that some of the findings are due to chance. The included trials in our study mostly have patients with prior anti-TNF agents as the primary biologic failure or intolerance, with insufficient data available regarding other biologics. Data regarding which prior anti-TNF was used by the patients were also unavailable, and it is unclear how relevant this may be to the outcomes observed here. Another limitation is that the clinical trials evaluated did not have routine endoscopy collected at postinduction which limits the conclusions that can be drawn regarding endoscopic outcomes in patients with biologic intolerance vs failure. Lastly, the applicability of our study findings and the relevance of prior biologic failure or intolerance on outcomes when treated with small molecules, such as JAK inhibitors and S1P modulators, is uncertain. This study is intended to generate hypotheses, and our results should be interpreted as potential associations rather than confirming a hypothesis.

## 5. Conclusion

In conclusion, our study provides valuable insights into the differential treatment responses among IBD patients based on their prior biologic exposure. Prior biologic intolerance may be associated with favorable treatment responses as compared to prior biologic failure. These results suggest that future clinical trials should more rigorously differentiate prior biologic failure from intolerance in the eligibility criteria and for stratification when randomizing patients into clinical trials of IBD.

## Supplementary Data

Supplementary data are available online at *ECCO-JCC* online.

jjae151_suppl_Supplementary_Tables

## Data Availability

This publication (Vivli protocol #00009332) is based on research using data from Takeda Inc. that has been made available through Vivli, Inc. Vivli has not contributed to or approved, and is not in any way responsible for, the contents of this publication. This study, carried out under YODA Project #2023-5300, used data obtained from the Yale University Open Data Access Project, which has an agreement with JANSSEN RESEARCH & DEVELOPMENT, L.L.C. The interpretation and reporting of research using this data are solely the responsibility of the authors and does not necessarily represent the official views of the Yale University Open Data Access Project or JANSSEN RESEARCH & DEVELOPMENT, L.L.C.
