# Supplementary material for: Outcomes of Patients With Prior Biologic Intolerance Are Better Than Those With Biologic Failure in Clinical Trials of Inflammatory Bowel Disease
Source: J Crohns Colitis. 2024 Sep 20;19(3):jjae151. doi: 10.1093/ecco-jcc/jjae151 (PMC11945295; doi:10.1093/ecco-jcc/jjae151)
Supplement: jjae151_suppl_Supplementary_Tables [file jjae151_suppl_supplementary_tables.docx]

**Supplementary Table 1. Baseline characteristics for UC patients stratified by treatment**

|  | Vedolizumab (n=712) | | | Ustekinumab (n=466) | | |
| --- | --- | --- | --- | --- | --- | --- |
| Variable | Prior failure of 1 biologic (n=233) | Prior Intolerance of 1 biologic (n=58) | Biologic Naïve (n=421) | Prior failure of 1 biologic (n=94) | Prior Intolerance of 1 biologic (n=6) | Biologic Naïve (n=366) |
| Age in years, mean (SD) | 40.1 (13.4) | 39.9 (14.4) | 40.7 (13.7) | 40.9 (15.4) | 37.5 (13.4) | 40.3 (12.4) |
| Sex Male, n (%) | 151 (64.8) | 23 (39.7) | 253 (60.1) | 67 (71.2) | 4 (66.7) | 211 (57.7) |
| Race White, n (%) | 210 (90.1) | 52 (89.7) | 341 (81.0) | 88 (93.6) | 4 (66.7) | 274 (74.9) |
| Smoking, n (%) | 23 (9.9) | 8 (13.8) | 75 (17.8) | 4 (4.3) | 3 (50.0) | 73 (19.9) |
| Disease Duration Years, median (IQR) | 4.9 (5.3) | 5.9 (5.1) | 3.8 (6.5) | 4.8 (6.4) | 7.2 (4.3) | 4.2 (8.5) |
| CRP mg/L, median (IQR) | 4.5 (11.3) | 8.8 (7.4) | 5.1 (10.1) | 4.6 (10.1) | 7.9 (7.7) | 5.1 (12.8) |
| Albumin g/L, mean (SD) | 38.8 (5.9) | 41.7 (6.4) | 39.6 (4.3) | 37.3 (6.3) | 39.9 (7.5) | 40.1 (5.7) |
| Total Mayo Score, mean (SD) | 8.9 (2.1) | 8.1 (1.9) | 8.5 (2.1) | 8.5 (1.9) | 8.1 (1.8) | 8.5 (1.7) |
| Endoscopic Mayo Score 3, n (%) | 163 (70.0) | 33 (56.9) | 295 (70.1) | 62 (66.0) | 2 (33.3) | 181 (49.5) |

**Supplementary Table 2. Baseline characteristics for CD patients stratified by treatment**

|  | Vedolizumab (n=967) | | | Ustekinumab (n=472) | | |
| --- | --- | --- | --- | --- | --- | --- |
| Variable | Prior failure of 1 biologic (n=458) | Prior Intolerance of 1 biologic (n=161) | Biologic Naïve (n=348) | Prior failure of 1 biologic (n=192) | Prior Intolerance of 1 biologic (n=64) | Biologic Naïve (n=216) |
| Age in years, mean (SD) | 36.1 (13.4) | 38.6 (12.5) | 34.8 (11.5) | 35.1 (9.9) | 37.5 (14.4) | 37.4 (14.2) |
| Sex Male, n (%) | 192 (41.9) | 37 (23.0) | 146 (42.0) | 90 (46.9) | 17 (26.6) | 128 (59.3) |
| Race White, n (%) | 441 (96.3) | 153 (95.0) | 309 (88.8) | 173 (90.1) | 56 (87.5) | 184 (85.2) |
| Smoking, n (%) | 188 (41.0) | 52 (32.3) | 118 (33.9) | 108 (56.3) | 21 (32.8) | 50 (23.1) |
| Disease Duration Years, median (IQR) | 7.4 (6.6) | 8.4 (6.4) | 3.8 (4.3) | 8.1 (7.2) | 9.4 (5.5) | 5.1 (5.2) |
| CRP mg/L, median (IQR) | 9.2 (21.4) | 8.3 (20.4) | 9.3 (21.6) | 8.4 (16.7) | 9.2 (18.5) | 8.9 (21.5) |
| Albumin g/L, mean (SD) | 31.1 (6.4) | 32.5 (6.4) | 36.1 (6.3) | 34.4 (5.8) | 36.3 (5.6) | 35.4 (6.5) |
| CDAI Score, mean (SD) | 325.6 (74.5) | 327 (69.7) | 316.3 (68.9) | 329 (76.2) | 315.4 (46.8) | 309.5 (69.1) |

**Supplementary Table 3**. **Outcomes at post-induction among patients with ulcerative colitis treated with ustekinumab or vedolizumab in the UNIFI and GEMINI I studies stratified by non-response status**. P < 0.05 is significant

| Outcome | Prior failure of 1 biologic (n=327) | Primary non-response (n=157) | Secondary non-response (n=170) | Prior intolerance of 1 biologic (n=64) | p-value (primary vs. secondary vs. intolerance) |
| --- | --- | --- | --- | --- | --- |
| Post-induction clinical response* | 127/327 (38.8) | 64/157 (40.8) | 63/170 (37.1) | 35/64 (54.7) | 0.002 |
| Post-induction clinical remission** | 36/327 (11.0) | 17/157 (10.8) | 19/170 (11.2) | 16/64 (25.0) | <0.001 |
| Post-induction endoscopic improvement*** | 81/327 (24.8) | 36/157 (22.9) | 45/170 (26.5) | 26/64 (40.6) | 0.002 |

*Reduction in Mayo score by at least 3 points and a decrease of at least 30% from baseline, with an accompanying decrease in the rectal bleeding subscore of at least 1 point or an absolute rectal bleeding subscore of 0 or 1

** Mayo score of 2 or lower and no subscore higher than 1

***MES <=1

**Supplementary Table 4. Univariate and multivariate logistic regression for UC patients achieving clinical remission at post-induction.**

|  | Univariate | | Multivariate | |
| --- | --- | --- | --- | --- |
| Baseline Variable | **Odds Ratio** (95 % CI) | **P-value** | **Odds Ratio** (95 % CI) | **P-value** |
| Age | 0.99 (0.98-1) | 0.09 |  |  |
| Sex (Male) | 1.53 (1.14-2.05) | **0.005*** | 1.8 (1.12-2.89) | **0.015*** |
| Race (White) | 1.53 (1.09-2.15) | **0.015*** | N/A | 0.262 |
| Smoking | 1.71 (0.86-3.42) | 0.126 |  |  |
| Disease Duration | 1 (0.99-1.02) | 0.686 |  |  |
| Treatment (vedolizumab vs. ustekinumab) | 1.22 (0.90-1.66) | 0.196 |  |  |
| Biologic Failure | 0.476 (0.3-0.754) | **0.002*** | 0.34 (0.18-0.66) | **0.001*** |
| Biologic Intolerant | 2.09 (1.18-3.69) | **0.011*** | N/A | 0.516 |
| Bio Naive | 1.37 (0.94-2) | 0.105 |  |  |
| CRP | 0.99 (0.98-1.01) | 0.535 |  |  |
| Albumin | 1.05 (1.02-1.09) | **0.005*** | 1.09 (1.04-1.15) | **<0.001*** |
| Total Mayo Score | 0.69 (0.63-0.79) | **<0.001*** | 0.71 (0.61-0.82) | **<0.001*** |
| Endoscopic Mayo Score 3 | 0.49 (0.37-0.67) | **<0.001*** | N/A | 0.171 |

**Supplementary Table 5. Outcomes at week 8 among patients with ulcerative colitis treated with ustekinumab in the UNIFI study.** P < 0.05 is significant

| Outcome | Prior failure of 1 biologic (n=94) | Prior intolerance of 1 biologic (n=6) | Biologic naïve (n=366) | p-value (failure vs. intolerance) | p-value (3 groups) |
| --- | --- | --- | --- | --- | --- |
| Week 8 clinical response* | 45/94 (47.9) | 4/6 (66.7) | 192/366 (52.5) | 0.372 | 0.556 |
| Week 8 clinical remission** | 9/94 (9.6) | 1/6 (16.7) | 60/366 (16.4) | 0.575 | 0.254 |
| Week 8 endoscopic improvement*** | 15/94 (16.0) | 1/6 (16.7) | 93/366 (25.4) | 0.963 | 0.144 |

**Reduction in Mayo score by at least 3 points and a decrease of at least 30% from baseline, with an accompanying decrease in the rectal bleeding subscore of at least 1 point or an absolute rectal bleeding subscore of 0 or 1*

*** Mayo score of 2 or lower and no subscore higher than 1*

****MES <=1*

**Supplementary Table 6. Outcomes at week 6 among patients with ulcerative colitis treated with vedolizumab in the GEMINI study.** P < 0.05 is significant

| Outcome | Prior failure of 1 biologic (n=233) | Prior intolerance of 1 biologic (n=58) | Biologic naïve (n=421) | p-value (failure vs. intolerance) | p-value (3 groups) |
| --- | --- | --- | --- | --- | --- |
| Week 6 clinical response* | 82/233 (35.2) | 31/58 (53.5) | 224/421 (53.3) | 0.011 | <0.001 |
| Week 6 clinical remission** | 27/233 (11.6) | 15/58 (25.9) | 96/421 (22.9) | 0.006 | 0.001 |
| Week 6 endoscopic improvement *** | 66/233 (28.2) | 25/58 (43.1) | 192/421 (45.6) | 0.028 | <0.001 |

**Reduction in Mayo score by at least 3 points and a decrease of at least 30% from baseline, with an accompanying decrease in the rectal bleeding subscore of at least 1 point or an absolute rectal bleeding subscore of 0 or 1*

*** Mayo score of 2 or lower and no subscore higher than 1*

****MES <=1*

**Supplementary Table 7. Outcomes at week 6 among patients with Crohn’s disease treated with ustekinumab or vedolizumab in the UNITI 1 & 2 and GEMINI 2 studies stratified by non-response status.** P < 0.05 is significant

| Outcome | Prior failure of 1 biologic (n=650) | Primary non-response (n=370) | Secondary non-response (n=280) | Prior intolerance of 1 biologic (n=225) | Biologic naïve (n=564) | p-value (primary vs. secondary vs. intolerance) |
| --- | --- | --- | --- | --- | --- | --- |
| Week 6 clinical response* | 213/650 (32.8) | 130/370 (35.1) | 83/280 (29.6) | 77/225 (34.2) | 284/564 (50.4) | 0.463 |
| Week 6 clinical remission** | 100/650 (15.4) | 59/370 (15.9) | 41/280 (14.6) | 45/225 (20.0) | 174/564 (30.9) | 0.352 |

*Reduction in CDAI of at least 100 points from baseline

**CDAI <=150

**Supplementary Table 8. Univariate and multivariate logistic regression with CD patients achieving clinical remission at week 6.**

|  | Univariate | | Multivariate | |
| --- | --- | --- | --- | --- |
| Baseline Variable | **Odds Ratio** (95 % CI) | **P-value** | **Odds Ratio** (95 % CI) | **P-value** |
| Age | 1.01 (0.99-1.01) | 0.38 |  |  |
| Sex (Male) | 0.98 (0.73-1.32) | 0.909 |  |  |
| Race (White) | 2.19 (1.59-3.02) | **<0.001*** | N/A | 0.656 |
| Smoking | 1.05 (0.79-1.38) | 0.729 |  |  |
| Disease Duration | 0.99 (0.98-0.99) | **<0.001*** | 1.15 (1.01- 1.71) | **0.002*** |
| Treatment (ustekinumab vs. vedolizumab) | 1.91 (1.42-2.56) | **<0.001*** | 3.5 (1.77-6.06) | **<0.001*** |
| Biologic Failure | 0.491 (0.36-0.74) | **0.001*** | N/A | 0.893 |
| Biologic Intolerant | 0.74 (0.53-1.04) | **<0.001*** | N/A | 0.275 |
| CRP | 1.01 (1-1.01) | **0.033*** | 1.02 (1-1.02) | **<0.001*** |
| Albumin | 1.05 (1.02-1.08) | **<0.001*** | 1.07 (1.03-1.1) | **<0.001*** |
| CDAI | 0.99 (0.98-0.99) | **<0.001*** | 0.99 (0.98-0.99) | **<0.001*** |

**Supplementary Table 9. Outcomes at week 6 among patients with Crohn’s disease treated with Vedolizumab from GEMINI 2 study (n=967).** P < 0.05 is significant

| Outcome | Prior failure of 1 biologic (n=458) | Prior intolerance of 1 biologic (n=161) | Biologic naïve (n=348) | p-value (failure vs. intolerance) | p-value (3 groups) |
| --- | --- | --- | --- | --- | --- |
| Week 6 clinical response* | 144/458 (31.4) | 55/161 (34.2) | 168/348 (48.3) | 0.525 | <0.001 |
| Week 6 clinical remission** | 69/458 (15.1) | 32/161 (19.9) | 94/348 (27.0) | 0.155 | <0.001 |

*Reduction in CDAI of at least 100 points from baseline

**CDAI <=150

**Supplementary Table 10. Outcomes at week 6 among patients with Crohn’s disease treated with Ustekinumab from UNITI 1 & 2 (weight-based only) (n=472).** P < 0.05 is significant

| Outcome | Prior failure of 1 biologic (n=192) | Prior intolerance of 1 biologic (n=64) | Biologic naïve (n=216) | p-value (failure vs. intolerance) | p-value (3 groups) |
| --- | --- | --- | --- | --- | --- |
| Week 6 clinical response* | 51/192 (26.6) | 22/64 (34.4) | 116/216 (53.7) | 0.231 | <0.001 |
| Week 6 clinical remission** | 31/192 (16.2) | 13/64 (20.3) | 80/216 (37.0) | 0.444 | <0.001 |

*Reduction in CDAI of at least 100 points from baseline

**CDAI <=150

**Supplementary Table 11. Median (IQR) disease duration across biologic treatment categories in inflammatory bowel disease trials.** P < 0.05 is significant

|  | Prior failure of 1 biologic | Prior intolerance of 1 biologic | Biologic naïve | p-value (failure vs. intolerance) | p-value (3 groups) |
| --- | --- | --- | --- | --- | --- |
| UNIFI (UC) | 5.2 (4.2-6.5) | 5.7 (4.3-6.8) | 3.2 (1.1-8.5) | 0.114 | 0.082 |
| GEMINI 1 (UC) | 5.8 (4.9-6.8) | 4.9 (3.8-5.7) | 3.6 (1.7-5.4) | 0.187 | 0.099 |
| GEMINI 2 (CD) | 5.8 (2.0-14.0) | 7.6 (2.8-13.8) | 4.7 (1.4-11.0) | 0.120 | 0.011 |
| UNITI 1 & 2 (CD) | 7.3 (3.5-11.8) | 8.1 (3.6-12.4) | 3.9 (1.4-8.5) | 0.438 | <0.001 |
